# Gamma irradiation crosslinked fluorescent nanocarbon based biodegradable hydrogel for controlled release of antibiotics

**DOI:** 10.1371/journal.pone.0340351

**Published:** 2026-01-16

**Authors:** Rajeshwar Vodeti, Mokhtar Rejili, Venkata Ramana Singamaneni, Umme Hani, Farhat Fatima, Jeetendra Kumar Gupta, Patibandla Jahnavi, Ramenani Hari Babu, Sharuk L. Khan, Md. Faysal

**Affiliations:** 1 Department of Pharmaceutics, School of Pharmacy, Anurag University, Hyderabad, Telangana, India; 2 Department of Biology, College of Sciences, Imam Mohammad Ibn Saud Islamic University (IMSIU), Riyadh, Saudi Arabia; 3 Senior Scientist II, Cambrex, Analytical Research and Development, Charles City, Iowa, United States of America; 4 Department of Pharmaceutics, College of Pharmacy, King Khalid University (KKU), Abha, Saudi Arabia; 5 Department of Pharmaceutics, College of Pharmacy, Prince Sattam Bin Abdulaziz University, Al-kharj, Saudi Arabia; 6 Department of Pharmacology, Institute of Pharmaceutical Research, GLA University, Mathura, Uttar Pradesh, India; 7 Wishmen Lifesciences Pvt Ltd, Banjara Hills, Hyderabad, Khairatabad, Telangana, India; 8 Department of Pharmacy Practice, Teerthanker Mahaveer College of Pharmacy, Teerthanker Mahaveer University, Moradabad, India; 9 Department of Pharmaceutical Chemistry, N.B.S Institute of Pharmacy, Ausa, Latur, Maharashtra, India; 10 Department of Pharmacy, Faculty of Health and Life Sciences, Daffodil International University, Dhaka, Bangladesh; Maulana Abul Kalam Azad University of Technology West Bengal, INDIA

## Abstract

Controlled and sustained antibiotic delivery is critical for combating antimicrobial resistance while minimizing side effects. Herein, a novel biodegradable hydrogel system, synthesized via gamma irradiation, incorporating fluorescent carbon dots (CDs) as multifunctional nano-crosslinkers, has been reported. The CDs, prepared from sustainable bio-precursors, reinforced the polymer network and enhanced the mechanical stability and swelling behavior, while simultaneously serving as intrinsic fluorescent probes for potential real-time monitoring of degradation and drug release. Thorough characterization revealed consistent morphology, adjustable biodegradability, and enhanced rheological characteristics. Drug release investigations demonstrated a diffusion-controlled mechanism, wherein the integration of CD diminished the cumulative antibiotic release from approximately 70% to approximately 40%, thereby facilitating precise regulation of release kinetics. The single-step gamma irradiation method facilitates concurrent crosslinking and sterilization, providing an efficient and scalable production strategy. This study presents a multifunctional hydrogel platform that integrates sustainable nanomaterials, regulated drug administration, and real-time monitoring, thereby facilitating the development of advanced theragnostic systems.

## Introduction

The escalating global crisis of antimicrobial resistance poses a formidable challenge to modern medicine, undermining the efficacy of conventional antibiotic therapies, and necessitating the development of advanced drug delivery platforms [1,2]. Traditional oral or intravenous antibiotic delivery generally leads to poor pharmacokinetics, with plasma concentrations varying below the minimal inhibitory concentration [3]. This subtherapeutic exposure nurtures bacterial adaptation and resistance, whereas peak concentrations may cause systemic toxicity [4,5]. Controlled-release systems are a paradigm shifting method for maintaining a localized and sustained therapeutic dosage of antimicrobial medicines at the infection site for a longer period [6]. This approach not only enhances treatment efficacy but also minimizes systemic side effects and reduces dosing frequency, thereby improving patient compliance [7–9]. Among the various matrices explored for this purpose, hydrogels, which are three-dimensional hydrophilic polymer networks capable of imbibing large quantities of water, have garnered significant attention because of their exceptional biocompatibility [10], tunable physicochemical properties [11,12], and structural resemblance to natural extracellular matrices [13].

The ideal hydrogel for biomedical applications, particularly for controlled drug release, must satisfy a stringent set of criteria: high aqueous absorbency for sufficient drug loading [14], mechanical robustness to maintain structural integrity, and, crucially, biodegradability to obviate the need for surgical removal after fulfilling its purpose [15]. Historically, hydrogels have been fabricated using chemical crosslinkers (e.g., formaldehyde or glutaraldehyde) or photopolymerization techniques [16]. However, these methods often introduce cytotoxic residues or require photoinitiators and UV light, which can be detrimental to the encapsulated bioactive molecules [17–19]. Gamma irradiation-induced crosslinking is a better “green” synthesis method. This solvent-free, initiator-less approach controls the crosslinking density and network design by adjusting the irradiation dose [20]. For instance, recent studies have demonstrated that gamma-irradiation-crosslinked hydrogels based on poly(vinyl alcohol) (PVA) and poly(ethylene glycol) (PEG) exhibit excellent gel fractions, swelling ratios, and mechanical properties while maintaining biocompatibility [21–23]. The integration of multifunctional nanomaterials presents a powerful avenue to engineer “smart” hydrogels with enhanced and novel properties [24].

Carbon dots (CDs), a nascent class of quasi-zero-dimensional carbon nanomaterials, have recently been exploited in the research landscape because of their compelling attributes, including excellent water solubility [25], robust chemical inertness [26], low toxicity [27], and biocompatibility [28–30]. In addition to their fluorescence properties, which are useful for bioimaging and tracking, CDs have many surface functional groups (e.g., carboxyl, hydroxyl, and amine) that make them ideal nanoplatforms for drug conjugation and multifunctional crosslinking hubs in polymers [31]. Their incorporation into a hydrogel network can profoundly influence swelling behavior, mechanical strength, and degradation kinetics [32,33]. More crucially, CDs’ complex surface chemistry of CDs facilitates strong contact with polymer chains and drug molecules, enabling complicated drug release mechanisms driven by infection site environmental stimuli, such as pH or enzyme activity. Researchers have improved the drug-loading capacity and controlled release characteristics of hydrogels for cancer therapy and antimicrobials using CDs from sustainable bio-precursors [34,35].

Despite these advancements, the in situ integration of fluorescent CDs into gamma-irradiated biodegradable hydrogels for antibiotic delivery remains underexplored [36]. This gap is significant given the potential synergies: CDs could provide real-time tracking capabilities via their innate fluorescence, while the gamma irradiation process ensures a sterile, contaminant-free network with precisely controlled crosslinking [37]. Similarly, studies on poly(N-vinyl pyrrolidone) (PVP) nanogels synthesized using gamma irradiation underscore the critical role of the saturation atmosphere and dose rate in controlling the particle size and crosslinking density [38]. These findings reinforce the potential of gamma irradiation in crafting advanced drug delivery systems but also emphasize the need for optimization to balance gel fraction, swelling, and drug release kinetics [39].

We rationally designed, synthesized, and evaluated a new multifunctional hydrogel structure for controlled antibiotic release. Our innovative synthesis method combines in situ polymerization and crosslinking of a biodegradable polymer network with luminous carbon dots inserted at its core. The CDs were synthesized from sustainable bio-precursors, following green chemistry principles, and served two purposes: as multifunctional nano-crosslinkers that enhance polymer network formation and mechanical and rheological properties, and as intrinsic fluorescent probes that allow real-time hydrogel degradation and drug release monitoring. Gamma irradiation solidifies and sterilizes the architecture in a single process, thus creating a pure biomaterial. We expect this CD-integrated hydrogel to have a higher swelling capacity, mechanical resilience, and programmable biodegradation, which will help control the release kinetics of model antibiotics. The shape, swelling ratio, mechanical strength, and degradation profile of the produced CDs and composite hydrogels were characterized in this study. Simulated physiological conditions were used to study the loaded antibiotic release kinetics. It has been shown that the approach works against important bacterial strains, linking the material’s characteristics to its biological performance. This study presents a versatile and resilient platform technology that combines nanomaterial science, polymer chemistry, and pharmaceutical technology. It tackles the urgent issue of regulated antibiotic delivery to prevent AMR and provides new options for theranostic devices that integrate therapy and monitoring into a single biodegradable implant.

## Materials and methods

### Materials

Fresh onions (*Allium cepa*) were procured from a local market in Mumbai, India and used as the natural precursor for the synthesis of fluorescent carbon dots. Guar gum (GG, food grade), 3-(trimethoxysilyl)propyl methacrylate (TMSPM, ≥ 98% purity), and other analytical-grade reagents were purchased from Sigma-Aldrich (Merck KGaA, Darmstadt, Germany) and used without further purification. Ciprofloxacin, a model antibiotic drug employed for controlled release studies, was kindly provided as a gift by Cipla Ltd. (Mumbai, India). Gamma irradiation was performed at a certified irradiation facility in India to achieve crosslinking of the hydrogel system. All aqueous solutions were prepared using double-distilled water. Standard buffer solutions of varying pH were prepared according to established protocols (Sigma-Aldrich, Merck KGaA, Darmstadt, Germany) and were used for swelling, stability, and drug release experiments.

### Synthesis of carbon dots (ONCDs)

**Fig 1:**
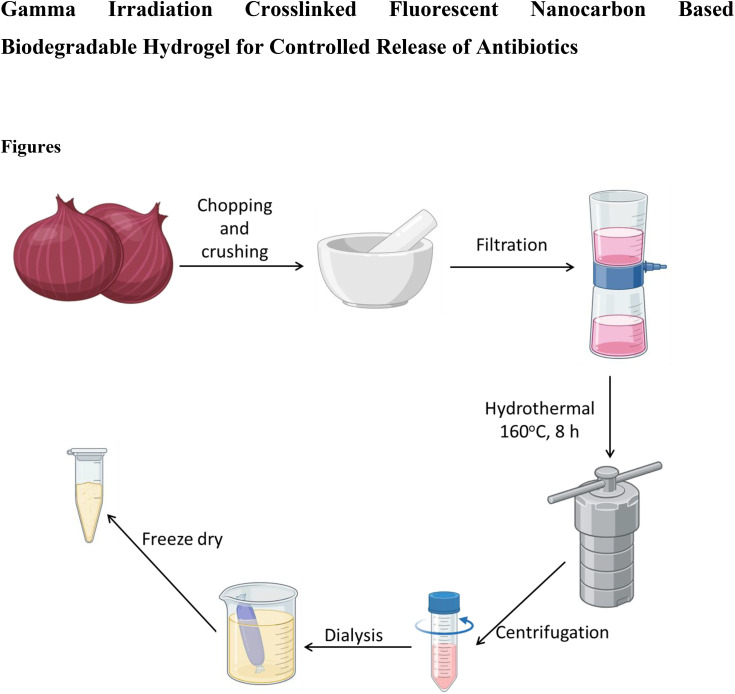
Synthesis of carbon dots from onion (ONCDs) by hydrothermal process.

Fresh onions were peeled, rinsed with distilled water, and chopped; 50 g of tissue was triturated in a mortar with 50 mL of distilled water to obtain a homogeneous slurry. The mash was gravity-filtered through a qualitative filter paper (Whatman No. 1), yielding ~40 mL of pale pink filtrate. Thirty milliliters of filtrate were transferred to a 50 mL Teflon-lined stainless-steel autoclave and hydrothermally treated at 160 °C for 8 h. After naturally cooling to room temperature, the reaction mixture was centrifuged at 10000 rpm for 10 min to remove insoluble residues. The supernatant was dialyzed against distilled water using a 1 kDa MWCO membrane for 24 h with 3–4 water changes to remove salts and low-molecular-weight impurities. The dialyzed dispersion was frozen and lyophilized to obtain onion-derived carbon dots (ONCDs) as a light brown powder (typically 60–80 mg from a 30 mL precursor). The Dry ONCDs were stored in the dark at 4 °C until use. Fig 1 shows the complete synthesis procedure for the ONCDs.

### Synthesis of ONCDs based hydrogels (HGCD)

ONCD-based hydrogels (HGCD) were fabricated via in situ polymerization and subsequent gamma-irradiation-induced crosslinking. Briefly, guar gum (1.0 g) was dispersed in 20 mL of double-distilled water under continuous stirring at 60 °C until a homogeneous viscous solution was obtained. ONCDs (50 mg, obtained as described above) were dispersed in 5 mL of water by ultrasonication for 10 min and then added dropwise to the guar gum solution under stirring. To enhance the interaction between the polysaccharide backbone and nanodots, 3-(trimethoxysilyl) propyl methacrylate (TMSPM, 100 µL) was added to the mixture and stirred for an additional 30 min. The resulting solution was cast into sterile glass vials (5 mL each) and subjected to gamma irradiation at a total dose of 25 kGy, using a 60Co source at a certified irradiation facility in India. Irradiation promotes free-radical polymerization and effective crosslinking of the ONCD–guar gum–TMSPM network, leading to the formation of a stable fluorescent hydrogel (HGCD). The concentration of CDs was varied at 0.1, 0.3, and 0.7%, and the resulting hydrogels were designated HGCDs1, HGCDs2, and HGCDs3, respectively. The obtained hydrogels were washed thoroughly with distilled water to remove any unreacted components and then stored at 4 °C for further characterization and drug loading studies. Gamma irradiation of the hydrogel samples was carried out using a Cobalt-60 source (specify model and facility) at a total dose of 25 kGy and a dose rate of approximately 1.0 kGy·h ⁻ ¹ under a nitrogen atmosphere to prevent oxidative degradation. The samples were sealed in airtight polyethylene pouches before irradiation and maintained at ambient temperature throughout the process. The selected dose of 25 kGy was based on the ISO 11137 standards, which recommend this level for achieving a Sterility Assurance Level (SAL) of 10 ⁻ ⁶ while effectively promoting polymer crosslinking. This dose was optimized to ensure sufficient network formation without excessive chain scission or degradation of biodegradable polymers or fluorescent carbon dots. The nitrogen environment further minimized the oxidative chain breakage and preserved the fluorescence and mechanical integrity of the resulting hydrogel. The chosen dose rate allowed for uniform energy deposition and stable free-radical generation, supporting consistent crosslinking across the bulk material. Similar parameters have been reported in previous studies on the sterilization and structural reinforcement of polymer–nanocomposite hydrogels.

### Ciprofloxacin loading and release studies

Ciprofloxacin loading into the ONCD-based hydrogel (HGCD) was achieved by passive diffusion. Preformed hydrogel discs (~0.5 g each, wet weight) were equilibrated in 10 mL of ciprofloxacin aqueous solution (1 mg mL ⁻ ¹ in phosphate-buffered saline, PBS, pH 7.4) at 25 °C under gentle shaking (100 rpm) for 24 h. After loading, the drug-entrapped hydrogels were removed, blotted to eliminate excess surface solution, and briefly rinsed with PBS to remove the unbound drug. The amount of ciprofloxacin (8 mg/g of hydrogel) incorporated was quantified by measuring the residual drug concentration in the supernatant at 276 nm using a UV–Vis spectrophotometer (Shimadzu, Japan), and the loading efficiency was calculated. For release studies, the ciprofloxacin-loaded HGCDs were transferred into 20 mL of PBS (pH 7.4) and maintained at 37 °C under gentle shaking. At predetermined time intervals (0.5, 1, 2, 4, 6, and 8 h), 2 mL of the release medium was withdrawn and replaced with an equal volume of fresh buffer to maintain the sink conditions. The ciprofloxacin concentration in each aliquot was determined spectrophotometrically at 276 nm and the cumulative release (%) was calculated and plotted as a function of time.

### Characterizations

The structural and physicochemical properties of the synthesized hydrogel nanocomposites were systematically characterized using advanced analytical techniques. Fourier-transform infrared spectroscopy (FTIR) analysis was conducted using a PerkinElmer Spectrum Two spectrometer in the range of 4000–400 cm ⁻ ¹ with 4 cm ⁻ ¹ resolution to identify functional groups and chemical interactions [40–43]. Crystallinity was examined by X-ray diffraction (XRD) measurements on a Rigaku SmartLab diffractometer using Cu Kα radiation (λ = 1.5406 Å) at a scanning rate of 2° min ⁻ ¹ over the 2θ range of 5–80° [44–46]. Optical properties were evaluated by photoluminescence (PL) spectroscopy using a Horiba Fluorolog-3 spectrofluorometer, and UV-Vis absorption spectra were recorded on a Shimadzu UV-2600 spectrophotometer in the 200–800 nm range [47,48]. Thermal stability was assessed by thermogravimetric analysis (TGA) on a TA Instruments Q50 analyzer under nitrogen atmosphere with a heating rate of 10°C min ⁻ ¹ from room temperature to 800°C. Morphological characterization was performed using field-emission scanning electron microscopy (FESEM; Carl Zeiss Sigma 300 VP) operated at 5 kV. The mechanical properties were evaluated through tensile and compression tests using an Instron 5966 universal testing machine equipped with a 500 N load cell at a crosshead speed of 5 mm/min. In vitro cytotoxicity was assessed via the MTT assay using a BioTek Synergy H1 microplate reader to measure the absorbance at 570 nm. All characterizations were performed under ambient conditions (25°C, 60% RH) unless otherwise specified.

### Measurement of degree of crosslinking

The desiccated produced films were contained in glass ampoules and irradiated in the presence of oxygen at doses ranging from 1 to 15 kGy, with a dose rate of 6.79 kGy h^−1^. After irradiation, the films were subjected to ethanol rinsing under magnetic agitation for 24 h, with solvent replacement occurring every 3 h to eliminate unreacted monomers. This was followed by a 24 h water wash to remove the uncross-linked XG. The samples were ultimately dried in a vacuum oven at 50°C until a consistent weight was obtained.

The crosslinking percentage (XC) was determined using Equation 1 as shown below:


$$\text{XC}=({\text{w}}_{\text{f}}/{\text{w}}_{\text{o}})\times \text{100}$$ (1)

where wf is the dry weight of the crosslinking film after extraction and wo is the initial weight of the dried gel.

### Statistical analysis

All experiments, including swelling, mechanical testing, and drug release studies, were conducted in triplicate (n = 3), and the results are presented as mean ± standard deviation. Statistical significance was evaluated using one-way ANOVA followed by Tukey’s post hoc test with p < 0.05, considered significant.

## Results and discussions

### Synthesis and characterizations of ONCDs

**Fig 2:**
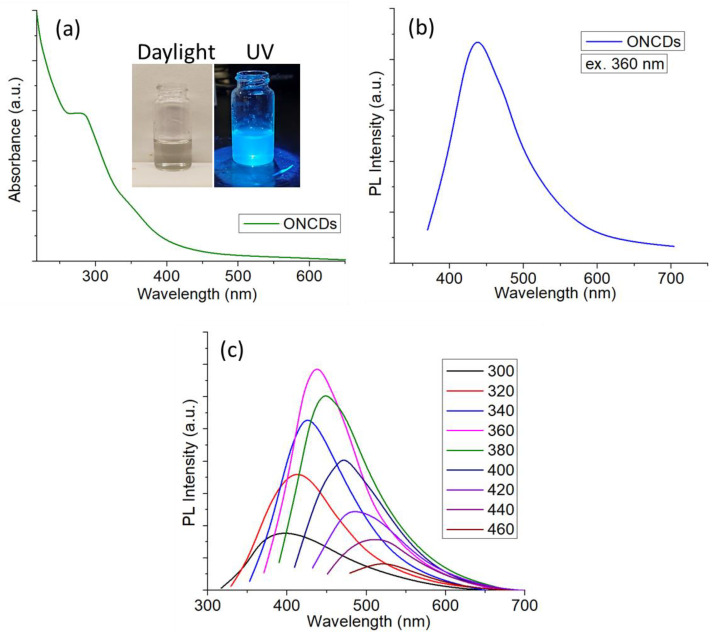
(a) UV-vis spectrum of ONCDs in aqueous medium (inset: ONCDs under daylight and 365 nm UV exposure) (b) PL spectrum of ONCDs at 360 nm excitation (c) excitation dependent PL spectra of ONCDs.

Onion-derived carbon dots (ONCDs) were successfully synthesized via a hydrothermal carbonization route, as schematically illustrated in Fig 1. The optical properties of the as-prepared ONCDs were investigated to confirm their successful formation and photoluminescence behavior (Fig 2). The UV–vis absorption spectrum of ONCDs in aqueous dispersion (Fig 2a) displays a strong absorption band below 300 nm, which can be attributed to the π–π* transition of aromatic C = C bonds, indicative of a partially graphitic core structure. A weak shoulder around 330–360 nm is ascribed to the n–π* transitions of C = O or C–N surface groups, reflecting the abundance of heteroatom functionalization [49]. The inset photographs of ONCDs under daylight and 365 nm UV exposure clearly demonstrate their strong blue fluorescence, further supporting the successful formation of highly emissive carbon nanostructures. Photoluminescence (PL) measurements provided additional insights into the electronic structure and emission properties of ONCDs. At an excitation wavelength of 360 nm (Fig 2b), the ONCDs exhibited a prominent emission peak centered at ~440 nm, corresponding to bright blue fluorescence. Such emission is typical for carbon dots and is generally associated with a combination of intrinsic bandgap transitions and surface/edge defect states. To further probe the optical response, excitation-dependent PL spectra were recorded (Fig 2c). With increasing excitation wavelength from 300 to 460 nm, the emission maxima exhibited a progressive red shift accompanied by variations in intensity. This excitation-dependent behavior is a characteristic feature of carbon dots and arises from the multiple emissive states associated with different surface functional groups and particle size distributions. The highest emission intensity was observed at 360 nm excitation, which was selected as the optimal condition for subsequent applications. Together, these optical characterizations confirm that the hydrothermal treatment of onion extract yields stable, water-dispersible carbon dots with abundant surface functionalities and strong blue fluorescence. The excitation-tunable emission, combined with biocompatibility from the natural precursor, renders ONCDs highly suitable for incorporation into hydrogel matrices for drug delivery applications.

**Fig 3:**
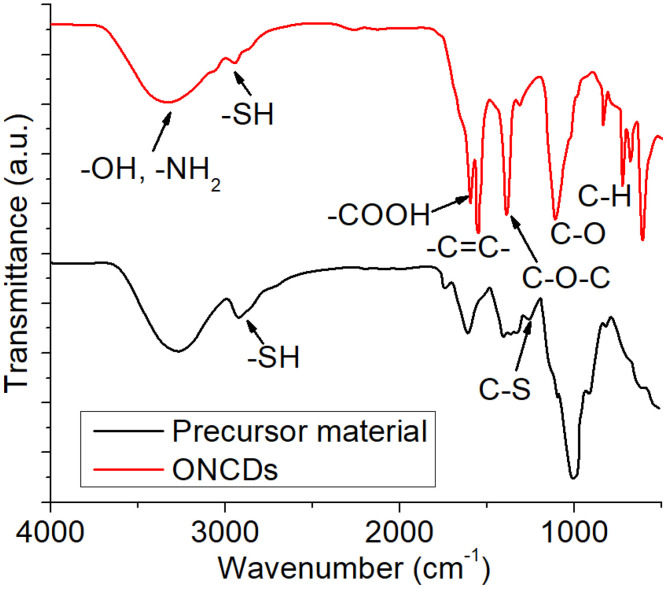
FTIR spectra of the precursor material of ONCDs and synthesized ONCDs.

The surface functional groups of the synthesized ONCDs were investigated using Fourier transform infrared (FTIR) spectroscopy, and the spectra were compared with those of the onion precursor material (Fig 3). The precursor spectrum exhibited broad absorption around 3400 cm ⁻ ¹, characteristic of O–H and N–H stretching vibrations from hydroxyl and amino groups, as well as distinct bands near 2550–2600 cm ⁻ ¹ attributed to thiol (–SH) stretching. Additional peaks at ~1720 cm ⁻ ¹ and ~1640 cm ⁻ ¹ correspond to C = O (carboxylic) and C = C stretching, respectively, while signals below 1200 cm ⁻ ¹ are associated with C–S vibrations, indicating the presence of sulfur-containing compounds inherent to the onion extract. After hydrothermal carbonization, the FTIR spectrum of the ONCDs (red curve) revealed notable changes, confirming successful carbonization and surface functionalization [50]. The broad band around 3400 cm ⁻ ¹ persisted, reflecting abundant O–H and N–H groups that contributed to the hydrophilicity and aqueous dispersibility. The distinct absorption at ~1720 cm ⁻ ¹ (C = O) and ~1620 cm ⁻ ¹ (C = C/C = N stretching) confirmed the presence of conjugated domains and residual heteroatom functionalities. Peaks at ~1380 cm ⁻ ¹ and ~1260 cm ⁻ ¹ were assigned to C–O and C–O–C stretching, suggesting the presence of oxygen-rich moieties on the ONCD surface. Additionally, the band near 2920–2850 cm ⁻ ¹ corresponds to the C–H stretching vibrations of sp³-hybridized carbons [51]. The absence of strong thiol-and C–S-related signals in ONCDs compared to the precursor indicates that the sulfur species were either decomposed or transformed during the hydrothermal process. These spectral features collectively confirm that ONCDs are decorated with multiple functional groups such as hydroxyl, amino, carboxyl, and epoxy groups, which not only stabilize the nanoparticles in an aqueous medium but also provide active sites for subsequent interactions with guar gum and ciprofloxacin in hydrogel formulations.

### Synthesis of nanocomposite hydrogels (HGCD)

**Fig 4:**
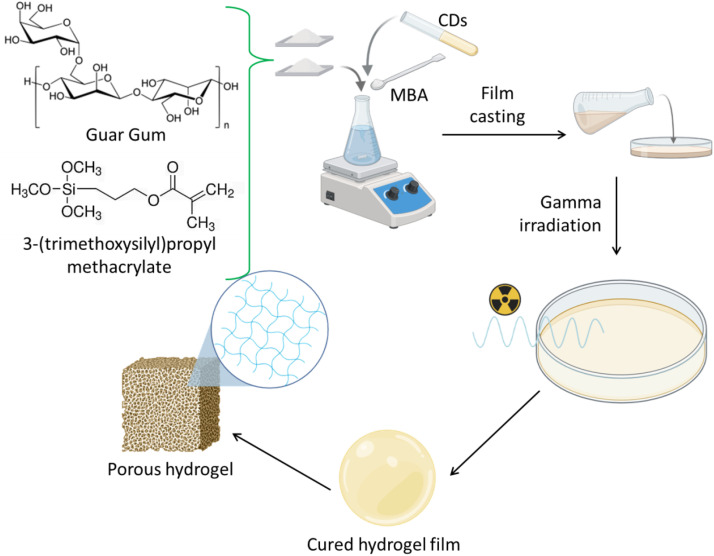
Schematic of the synthesis of nanocarbon based nanocomposite hydrogel.

The formation of ONCD-based nanocomposite hydrogels (HGCD) was achieved through a synergistic combination of polymer–nanofiller interactions and gamma irradiation crosslinking, as schematically illustrated in Fig 4. Guar gum, a naturally derived polysaccharide, serves as the primary hydrogel matrix owing to its hydrophilicity, biodegradability, and ability to form a three-dimensional polymeric network [52]. To improve the mechanical stability and introduce reactive sites for crosslinking, guar gum chains were functionalized with 3-(trimethoxysilyl)propyl methacrylate (TMSPM), which introduces silane groups capable of enhancing the polymer–polymer and polymer–nanodot interactions. The pre-synthesized ONCDs were uniformly dispersed in the guar gum–TMSPM mixture, allowing intimate interactions between the surface functional groups of ONCDs (–OH, –COOH, and–NH₂) and the polysaccharide matrix. The homogenous precursor solution was subsequently cast into molds to form the thin films. Subsequently, the films were exposed to gamma irradiation, which acted as a clean, initiator-free method to generate free radicals and promote efficient crosslinking between guar gum chains, TMSPM units, and embedded ONCDs. This process results in the formation of stable, mechanically reinforced, and fluorescent nanocomposite hydrogels. The final HGCDs exhibited a porous microstructure (as depicted in the schematic) that enhanced water uptake, swelling, and diffusion-mediated drug release while maintaining structural integrity. The incorporation of ONCDs within the polymeric framework not only imparted fluorescence, enabling potential optical tracking, but also contributed to improved crosslinking density through the surface functional groups [53]. Thus, this synthesis route successfully integrated renewable biopolymers, fluorescent nanocarbons, and irradiation-induced polymerization into a multifunctional hydrogel platform suitable for drug delivery applications.

### Effect of gamma irradiation dose on crosslinking of HGCDs

**Fig 5:**
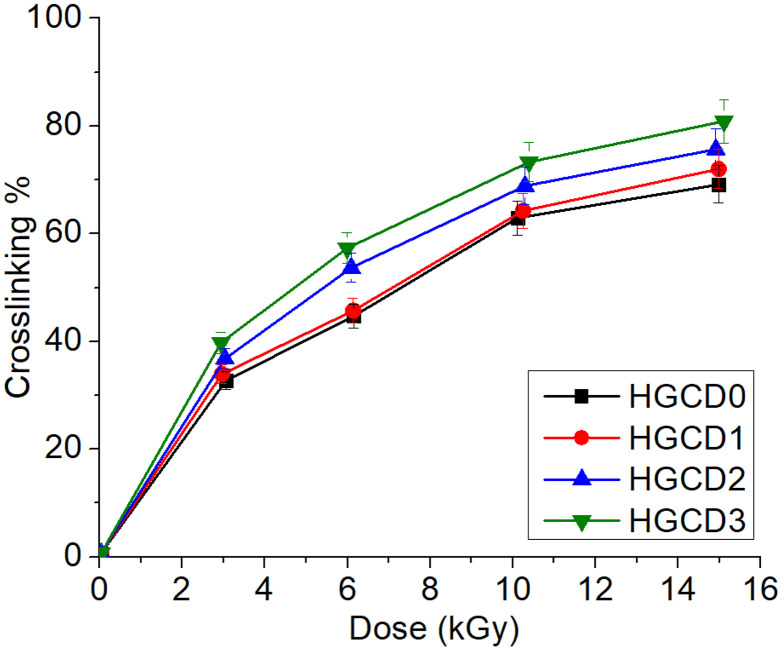
Degree of crosslinking as a function of dosage onto nanocomposite hydrogels.

The degree of crosslinking of the ONCD-based hydrogels (HGCDs) was evaluated as a function of gamma irradiation dose, and the results are presented in Fig 5. A clear dose-dependent increase in the crosslinking percentage was observed across all hydrogel formulations (HGCD0–HGCD3). At lower irradiation doses (2–4 kGy), the crosslinking percentage increased rapidly, indicating that the free radicals generated in the polymer matrix readily initiated intermolecular bonding between the guar gum chains, TMSPM, and ONCDs. Beyond 6 kGy, the crosslinking percentage continued to increase but at a relatively slower rate, eventually approaching saturation at higher doses (12–15 kGy) [54]. Among the formulations, HGCD3 consistently exhibited the highest crosslinking degree, followed by HGCD2 and HGCD1, whereas HGCD0 (control without ONCDs) showed the lowest values. This trend highlights the role of ONCDs as effective nano-crosslinking agents because of their abundant surface functionalities (–OH, –COOH, and–NH₂), which actively participate in radical-induced polymerization. The presence of ONCDs not only enhanced the crosslinking density, but also improved the efficiency of network formation under irradiation, as reflected by the higher crosslinking percentages compared to the pristine hydrogel. These results confirm that increasing the irradiation dose strengthens the hydrogel network by promoting more covalent linkages, while the incorporation of ONCDs further amplifies the crosslinking efficiency. Such improvements in crosslinking are expected to enhance the mechanical stability, swelling resistance, and sustained drug release performance of hydrogels, which are critical parameters for biomedical applications.

**Fig 6:**
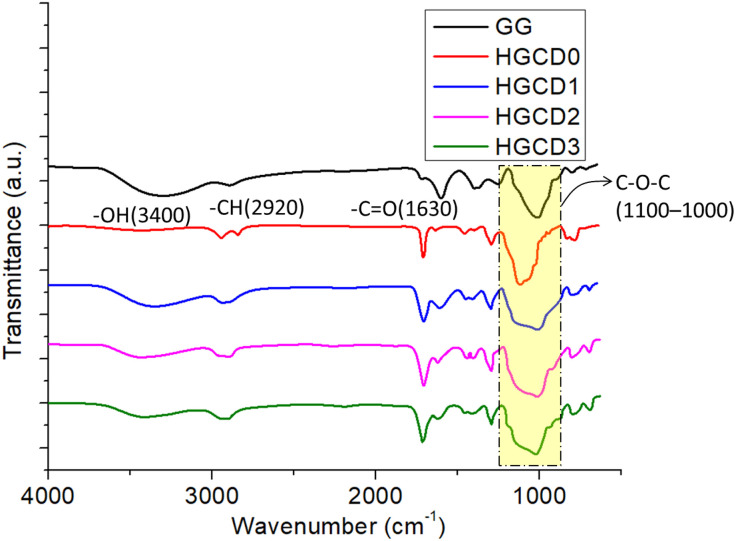
FTIR spectra of the pure polysaccharide (GG), unfilled hydrogel (without ONCDs) and nanocomposite (ONCDs loaded) hydrogels.

Fig 6 shows the FTIR spectra of pure guar gum (GG), the empty hydrogel (lacking ONCDs), and the ONCD-loaded nanocomposite hydrogel. The guar gum spectra exhibited a large absorption band at approximately 3400 cm ⁻ ¹, indicative of O–H stretching vibrations of hydroxyl groups, alongside signals near 2920 cm ⁻ ¹ associated with C–H stretching. The band at approximately 1630 cm ⁻ ¹ was attributed to absorbed water and C = O stretching from remaining uronic acid groups, whereas peaks in the range of 1100–1000 cm ⁻ ¹ indicated C–O–C stretching of the polysaccharide backbone [55]. In the case of the unfilled hydrogel (crosslinked without ONCDs), the characteristic absorption peaks of GG were retained, but noticeable changes were observed. A more intense band around 1720 cm ⁻ ¹ appeared, corresponding to ester linkages formed by the reaction between guar gum hydroxyl groups and the methacrylate moiety of TMSPM under gamma irradiation. This confirmed the successful incorporation of the silane crosslinker and the establishment of a stable hydrogel network. The ONCD-loaded nanocomposite hydrogel exhibited additional modifications in its FTIR profile [56]. The broad O–H/N–H stretching band around 3400 cm ⁻ ¹ became more intense, reflecting hydrogen-bonding interactions between the hydroxyl groups of guar gum and the surface functionalities of ONCDs (–OH, –COOH, and–NH₂). The enhancement of the ~ 1720 cm ⁻ ¹ band further indicates increased carbonyl contributions, likely from the ONCDs. Moreover, the C–O and C–O–C stretching vibrations (~1250–1050 cm ⁻ ¹) became more pronounced [57], confirming strong polymer–nanodot interactions and successful embedding of ONCDs within the hydrogel matrix.

**Fig 7:**
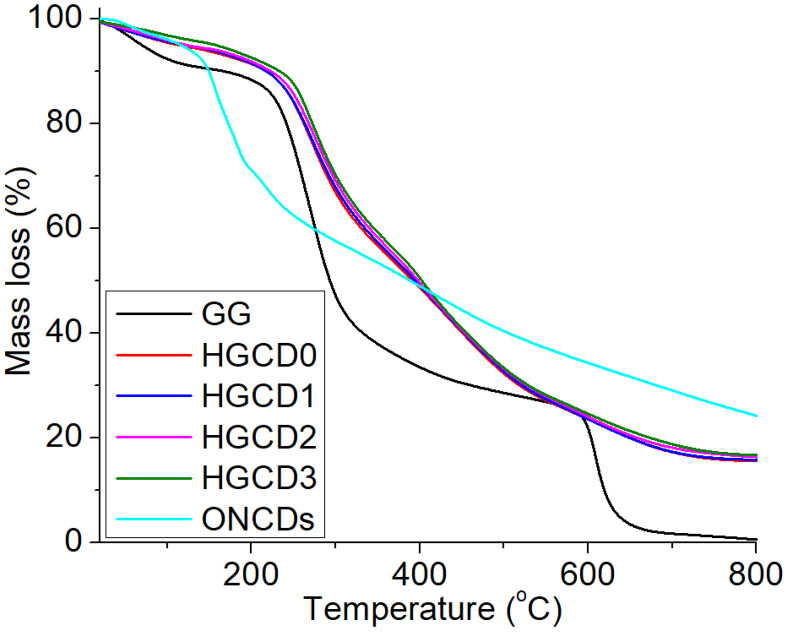
TGA analysis of ONCDs, unfilled hydrogel and impact of ONCDs onto hydrogels.

The thermal degradation behavior of ONCDs, the unfilled hydrogel (without ONCDs), and the ONCD-loaded nanocomposite hydrogel was studied by thermogravimetric analysis (TGA), as shown in Fig 7. The Pristine ONCDs exhibited an initial minor weight loss below 150 °C, attributed to the evaporation of adsorbed water and volatile surface groups. A major decomposition step was observed between 250–450 °C, corresponding to the degradation of carbonaceous frameworks and oxygenated surface functionalities, after which a stable carbonaceous residue remained. A three-step degradation profile was observed for the unfilled hydrogel. The first weight loss below 120 °C corresponded to the removal. The second stage (200–350 °C) reflects the breakdown of guar gum backbones and cleavage of the crosslinked silane (TMSPM) ester bonds. The third stage above 400 °C is associated with further degradation of the polymeric network into carbonaceous residues [58]. Interestingly, the ONCD-loaded nanocomposite hydrogel showed a noticeable shift in the onset of degradation toward higher temperatures compared to the unfilled hydrogel, indicating enhanced thermal stability. The incorporation of ONCDs delayed polymer decomposition, which can be attributed to strong hydrogen bonding and interfacial interactions between ONCDs’ functional groups (–OH, –COOH, and–NH₂) and the guar gum/silane matrix [59]. Furthermore, ONCDs likely act as thermal barriers and reinforcing fillers, restricting polymer chain mobility and slowing down thermal decomposition [60]. Hence, the TGA results confirm that ONCD incorporation not only improves the crosslinking density but also imparts a higher thermal resistance to the hydrogel network. Such thermal stability enhancement is advantageous for biomedical applications where structural integrity under physiological and sterilization conditions is essential.

**Fig 8:**
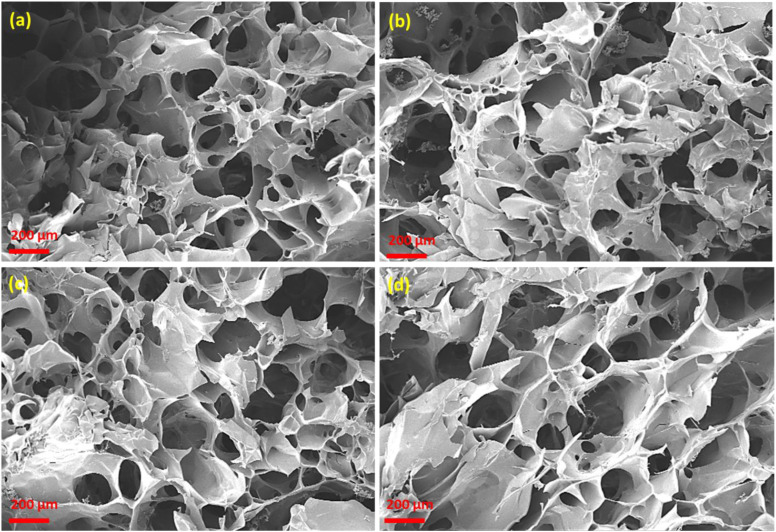
SEM images of the hydrogel showing porous morphology of (a) HGCD0 (b) HGCD1 (c) HGCD2 and (d) HGCD3.

The surface morphology of the hydrogels was examined by scanning electron microscopy (SEM), and representative images are shown in Fig 8. The unfilled hydrogel (HGCD0) exhibited a relatively smooth and less-defined porous structure, indicating a limited pore connectivity [61]. Upon the incorporation of ONCDs (HGCD1–HGCD3), a well-developed three-dimensional porous morphology was observed, with interconnected pores distributed uniformly throughout the hydrogel matrix. The porosity appeared to increase progressively with higher ONCD loading, as evident in HGCD2 and HGCD3, which displayed larger pore channels and enhanced interconnectivity compared with HGCD1 [62]. The ONCDs are likely responsible for modulating the network structure by promoting crosslinking interactions and preventing pore collapse during gelation. Such interconnected porous structures are advantageous for hydrogel applications in drug delivery because they can facilitate water absorption, diffusion of biomolecules, and controlled release of therapeutic agents [63]. Therefore, SEM analysis confirmed that ONCD incorporation not only reinforced the hydrogel network, but also significantly improved its porosity and internal architecture. SEM analysis showed that CD incorporation produced a more uniform and interconnected porous structure with average pore sizes ranging from 150 to 280 μm. This enhanced porosity likely contributed to the diffusion-controlled release of ciprofloxacin from the hydrogel.

**Fig 9:**
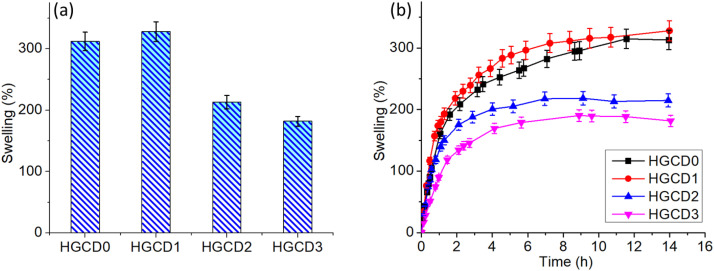
Water uptake behavior (swelling) of hydrogels and impact of ONCDs towards the gel matrices. Values represent mean ± SD (n = 3).

The swelling behavior of the hydrogel matrices was evaluated to understand the impact of ONCD incorporation on water uptake capacity. As shown in Fig 9a, the pristine hydrogel (HGCD0) exhibited a relatively high swelling ratio, which further increased in HGCD1 owing to the presence of a low ONCD content, which enhanced the hydrophilicity of the network [64]. However, HGCD2 and HGCD3 showed a progressive decrease in the swelling percentage. Owing to the larger ONCD loading, the polymeric matrix may have crosslinked more, reducing the water penetration and network growth. Swelling kinetics (Fig 9b) showed rapid water intake in the first several hours and a slower equilibrium stage [65]. Among all formulations, HGCD1 reached the highest swelling equilibrium, whereas HGCD3 displayed the lowest, confirming that excessive ONCD content hinders water uptake by tightening the gel structure.

**Fig 10:**
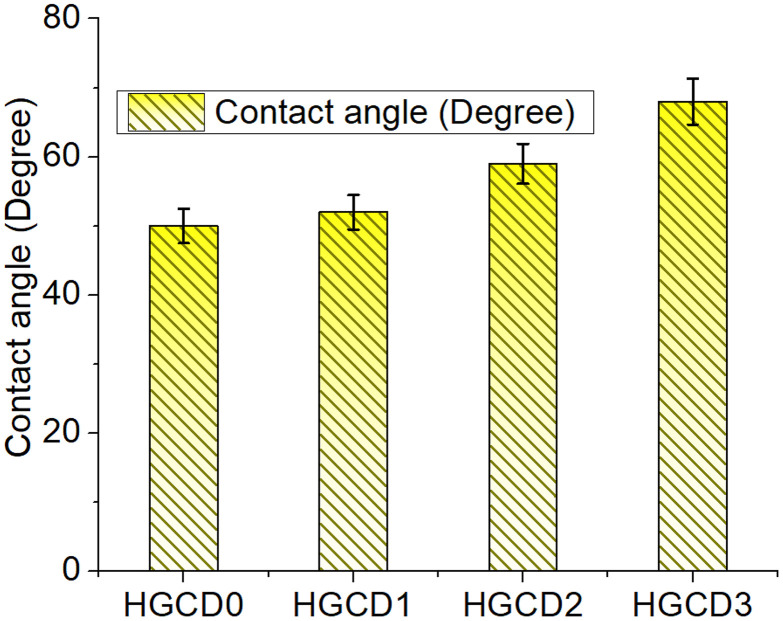
Contact angle measurement of the hydrogels showing surface wetting behavior of the hydrogels and impact of ONCDs. Values represent mean ± SD (n = 3).

The surface wettability of the hydrogels was further assessed using contact angle measurements (Fig 10). A progressive increase in the contact angle was observed from HGCD0 to HGCD3, indicating that a higher ONCD loading reduced the hydrophilic nature of the hydrogel surface. HGCD0 exhibited the lowest contact angle, suggesting better water affinity, whereas HGCD3 displayed the highest contact angle, reflecting its more hydrophobic character [66]. This trend is consistent with the swelling results, where a lower degree of water absorption corresponds to a higher contact angle [67]. Together, these findings demonstrate that controlled incorporation of ONCDs plays a significant role in tuning the swelling and wettability properties of hydrogels, which can be crucial for tailoring their performance in biomedical and environmental applications.

**Fig 11:**
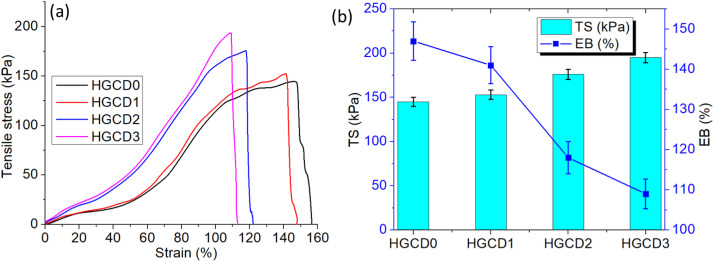
(a) Uniaxial tensile strength of the hydrogels (b) Tensile strength (TS) and elongation at break (EB) of the hydrogel after tensile stretching. Values represent mean ± SD (n = 3).

Hydrogel mechanical characteristics were studied to determine how the incorporation of ONCD affected structural integrity. Fig 11a shows that the hydrogel formulations had different uniaxial tensile stress–strain curves. The virgin hydrogel (HGCD0) has low tensile strength and stretchability, indicating a weaker polymeric network. ONCDs increased the mechanical performance, especially in HGCD1, which had the highest stress resistance and elongation before rupture. This enhancement can be attributed to the strong interfacial interactions between the ONCDs and the polymer chains, which act as additional physical crosslinking points and effectively dissipate stress under deformation. Quantitative analysis of the tensile strength (TS) and elongation at break (EB) (Fig 11b) further supported this observation. HGCD1 showed maximum values of both TS and EB, indicating an optimal reinforcement effect at lower ONCD concentrations [68]. However, increasing the ONCD content (HGCD2 and HGCD3) decreased TS and EB. At larger loadings, nanoparticle aggregation may disturb polymer matrix homogeneity and create stress concentration sites, reducing flexibility and strength. These findings show that ONCDs tune the hydrogel network by increasing the toughness and elasticity at intermediate concentrations and decreasing the mechanical performance owing to structural imbalance at high concentrations.

### Drug release behaviors

**Fig 12:**
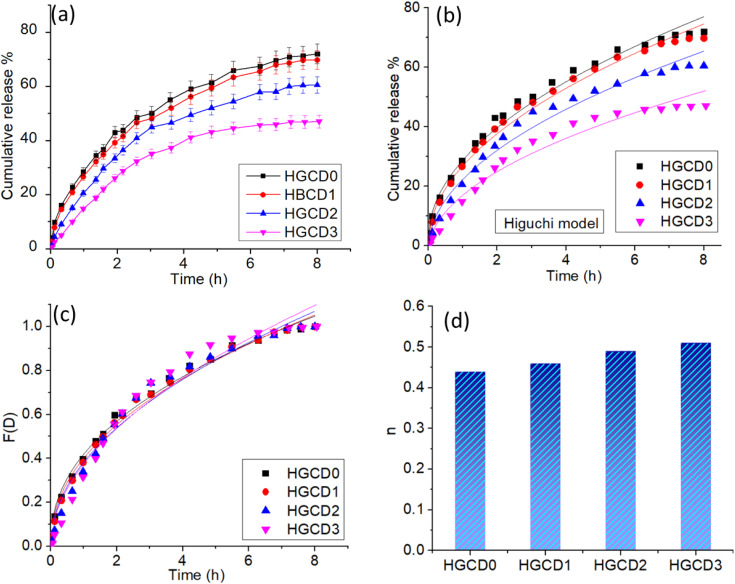
(a) Cumulative release profile of model drug molecules from the hydrogels and effect of CDs on release profiles (b) Higuchi model fittings of the cumulative release behavior of the hydrogels (c) Korsmeyer-Peppas model fittings of the hydrogels (d) Histogram plot of the Korsmeyer-Peppas exponent showing the values near to 0.5.


The release behavior of the model drug molecules from the HGCD hydrogels was systematically investigated to understand the role of ONCDs in modulating release kinetics. Fig 12a shows the cumulative release profiles of hydrogels with and without ONCD incorporation. Sustained release was observed in all cases; however, the ONCDs significantly reduced the release percentage, controlling the cumulative release from nearly 70% in pristine hydrogels to approximately 40% in ONCD-modified systems. This reduction highlights the ability of ONCDs to provide additional drug–matrix interactions (e.g., hydrogen bonding, π–π stacking, or electrostatic effects), which effectively retards drug diffusion and extends the release period. To further quantify the release mechanisms, the experimental data were fitted to two classical kinetic models: The Higuchi model [69] and Korsmeyer–Peppas model [70]. The Higuchi equation (Eq. 2) can be expressed as follows:

$${\text{Q}}_{\text{t}}={\text{k}}_{\text{H}}.{\text{t}}^{\text{0}.\text{5}}$$ (2)

where Q_t_ is the cumulative amount of drug released at time t and k_H_ is the Higuchi release constant. This model assumes Fickian diffusion of drug molecules through a porous polymeric matrix. As shown in Fig 12b, a good linear correlation is obtained, indicating that diffusion is the primary governing mechanism in the HGCD system. The slopes of the fitted lines further confirm the decreased diffusional rate constants in the presence of ONCDs, in line with the observed slower release profiles.

The Korsmeyer–Peppas model (Eq. 3) was applied to describe the release mechanism in more detail.


$$\text{F}(\text{D})=\frac{{\text{M}}_{\text{t}}}{{\text{M}}_{\infty }}={\text{k}}_{\text{KP}}.{\text{t}}^{\text{n}}$$ (3)

where M_t_/M_∞_ is the fraction of drug released at time t, k_KP_ is the kinetic constant, and n is the release exponent, indicating the release mechanism. For a slab-like geometry, 0.5n ≤ 0.5 corresponds to Fickian diffusion, 0.5 < n < 1 indicates anomalous (non-Fickian) transport, and n = 1 represents Case-II transport (relaxation-controlled release) [71]. Fig 12c shows the fitting results, while the histogram in Fig 12d illustrates the calculated n values, which were found to be close to 0.5, for all hydrogels. This finding confirms that the release process in HGCD is dominated by Fickian diffusion rather than polymer relaxation [72]. The presence of ONCDs reduces the effective diffusivity without altering the underlying release mechanism. These results demonstrate that the incorporation of ONCD into HGCD provides a tunable drug release platform by effectively lowering the release extent from ~70% to ~40% while maintaining a diffusion-controlled mechanism. The observed reduction in the cumulative ciprofloxacin release from ~70% to ~40% upon incorporation of ONCDs can be attributed to the multifunctional role of the CDs within the hydrogel network. The surface functional groups of ONCDs (–OH, –COOH, and–NH₂) are capable of forming hydrogen bonds and electrostatic interactions with ciprofloxacin molecules, effectively anchoring the drug and reducing its mobility. Additionally, CDs act as nano-crosslinkers, increasing the network density and decreasing the mesh size of the hydrogel, which further limits drug diffusion. This combination of specific drug-CDs interactions and structural reinforcement results in a more controlled and sustained release profile. Supporting evidence from zeta potential measurements and pH-dependent swelling studies confirms that ONCDs modulate both the hydrogel’s physicochemical environment and release kinetics, providing a mechanistic explanation for the enhanced drug retention. Such modulation is highly significant in drug delivery research because controlled and sustained release can minimize the dosing frequency, reduce side effects, and improve therapeutic efficiency. To confirm that the antibiotic retains its activity after release from the hydrogel, a microbroth growth inhibition assay was performed. Bacterial cultures of *E. coli* were incubated with the released drug and, for comparison, with fresh drug at equivalent concentrations in a 96-well plate for 12 h. Bacterial growth was monitored by measuring optical density at 600 nm (OD600). The control culture without drug reached an OD600 of 0.80, while cultures treated with fresh drug and released drug from the hydrogel reached OD600 values of 0.10 and 0.12, respectively, corresponding to growth inhibition of 86.57% and 85.82%. These results demonstrate that the released drug effectively inhibits bacterial growth to a level comparable to the fresh drug, confirming that the hydrogel system preserves the therapeutic activity of the antibiotic while enabling controlled and sustained release.

## Conclusions

This study successfully created and assessed a gamma-irradiation-cross-linked biodegradable hydrogel containing fluorescent carbon dots (CDs) for the controlled release of antibiotics. The multifunctional role of CDs, sourced from sustainable bio-precursors, was unequivocally demonstrated; they served as nano-crosslinkers that enhanced the polymer network and bolstered the structural and functional stability of the hydrogel. Thorough characterization validated the consistent CD integration, whereas swelling and degradation analyses emphasized adjustable water absorption and biodegradability. The mechanical and rheological assessments demonstrated improved stability and resilience, corroborating the robustness of the network. Drug release tests indicated a diffusion-controlled mechanism, with ONCD inclusion decreasing the cumulative release from approximately 70% to approximately 40%, facilitating more prolonged antibiotic delivery. These findings confirm that CD incorporation enhances hydrogel architecture and facilitates meticulous regulation of therapeutic efficacy, establishing this material as a viable platform for biodegradable antibiotic administration.

## Supporting information

S1 File Inclusivity in global research questionnaire tbe. (DOCX)
